# Angiogenic Imbalance in Preeclampsia: Profiling VEGF A, sFlt1, PlGF, and sFlt1/PlGF Ratios

**DOI:** 10.3390/ijms27052438

**Published:** 2026-03-06

**Authors:** Alexandru-Dan Assani, Lidia Boldeanu, Marius Bogdan Novac, Mohamed-Zakaria Assani, Isabela Siloși, Mihail Virgil Boldeanu, Anda Lorena Dijmărescu, Maria-Magdalena Manolea, Venera Cristina Dinescu, Constantin-Cristian Văduva

**Affiliations:** 1 Doctoral School, University of Medicine and Pharmacy of Craiova, 200349 Craiova, Romania; alexandruassani@gmail.com (A.-D.A.); mohamed.assani@umfcv.ro (M.-Z.A.); 2 Department of Microbiology, Faculty of Medicine, University of Medicine and Pharmacy of Craiova, 200349 Craiova, Romania; lidia.boldeanu@umfcv.ro; 3 Department of Anesthesiology and Intensive Care, Faculty of Medicine, University of Medicine and Pharmacy of Craiova, 200349 Craiova, Romania; 4 Department of Immunology, Faculty of Medicine, University of Medicine and Pharmacy of Craiova, 200349 Craiova, Romania; isabela_silosi@yahoo.com (I.S.); mihail.boldeanu@umfcv.ro (M.V.B.); 5 Department of Obstetrics and Gynecology, Faculty of Medicine, University of Medicine and Pharmacy of Craiova, 200349 Craiova, Romania; lorenadijmarescu@yahoo.com (A.L.D.); magdalena.manolea@umfcv.ro (M.-M.M.); cristian.vaduva@umfcv.ro (C.-C.V.); 6 Department of Health Promotion and Occupational Medicine, University of Medicine and Pharmacy of Craiova, 2 Petru Rares Str., 200349 Craiova, Romania

**Keywords:** preeclampsia, angiogenic imbalance, sFlt1, PlGF, sFlt1-to-PlGF ratio, VEGF A

## Abstract

Preeclampsia involves an angiogenic imbalance, but circulating vascular endothelial growth factor A (VEGF A) remains inconsistently described, particularly in relation to maternal adiposity. We studied 90 second-trimester pregnancies, 30 uncomplicated and 60 with preeclampsia, recording maternal body mass index (BMI) and gestational age at sampling. Serum soluble fms-like tyrosine kinase 1 (sFlt1), placental growth factor (PlGF), and VEGF A were measured by enzyme-linked immunosorbent assay (ELISA), and the sFlt1-to-PlGF ratio was calculated. Preeclampsia was associated with higher pre-pregnancy and pregnancy BMI, lower PlGF, and an approximately threefold higher sFlt1-to-PlGF ratio, while sFlt1 alone was only borderline higher. VEGF A was elevated in preeclampsia and rose across higher sFlt1-to-PlGF ratio categories, supporting the interpretation of VEGF A within the integrated sFlt1,PlGF axis rather than as an isolated signal.

## 1. Introduction

Preeclampsia is a pregnancy-specific hypertensive disorder defined by new-onset hypertension after 20 weeks of gestation, usually accompanied by proteinuria or maternal organ dysfunction. It affects about 2 to 8 percent of pregnancies worldwide and remains a leading cause of maternal and perinatal morbidity and mortality. Recent global estimates suggest that hypertensive disorders, including preeclampsia, account for around 17 percent of maternal deaths, with tens of thousands of women dying each year, especially in low-resource settings [1,2,3].

Clinically, preeclampsia is heterogeneous. It ranges from late-onset disease at term with relatively mild features to early-onset forms associated with fetal growth restriction, preterm delivery, and significant maternal complications. Women who survive preeclampsia have an increased long-term risk of cardiovascular disease, stroke, and diabetes, while their offspring are more likely to be born preterm and to develop cardiometabolic disease later in life [4,5,6]. Women with gestational diabetes mellitus (GDM) face a heightened risk of preeclampsia and gestational hypertension. Also, chronic endometritis (CE) is an inflammatory condition of the endometrium. It involves several local components, such as leukocytes, cytokines, immunoglobulins, and other factors that mediate the immune response and endometrial growth and endometrial inflammation that appear to alter the physiological mechanisms of oocyte fertilization. Placental dysfunction mediated by oxidative stress, inflammatory responses, and endothelial dysregulation contributes to elevated vascular resistance and hypertensive complications [7,8,9,10,11].

Maternal adiposity is one of the most common clinical risk factors for preeclampsia, and risk increases across the body mass index (BMI) distribution [12,13]. Because placental growth factor (PlGF), soluble fms-like tyrosine kinase 1 (sFlt1), and the sFlt1,PlGF ratio are used in screening and triage frameworks, BMI-related shifts in baseline angiogenic marker levels can affect risk stratification and the interpretation of fixed cutoffs, particularly in women with obesity [14,15]. Consistent with this, higher early pregnancy BMI has been associated with altered sFlt1 and PlGF trajectories, and BMI is recognized as a determinant of first-trimester PlGF in population studies, supporting the need to consider BMI when interpreting angiogenic profiles [15,16,17].

Current concepts of pathophysiology place placental malperfusion and syncytiotrophoblast stress at the center of the disorder. Inadequate remodeling of the uterine spiral arteries leads to placental ischemia and release of antiangiogenic and inflammatory factors into the maternal circulation. Among these, sFlt1 acts as a soluble form of the VEGF receptor 1 and binds circulating vascular endothelial growth factor A (VEGF A) and PlGF, reducing their bioavailability for endothelial receptors. This angiogenic imbalance contributes to systemic endothelial dysfunction, hypertension, proteinuria and end-organ injury [18,19,20,21].

Circulating angiogenic markers have therefore emerged as attractive biomarkers for the diagnosis and short-term prediction of preeclampsia. Low PlGF and high sFlt1 levels, and in particular an elevated sFlt1/PlGF ratio, reflect the degree of angiogenic imbalance and correlate with disease severity and adverse outcomes [22,23,24,25]. Research from large studies like PROGNOSIS has demonstrated that an sFlt1/PlGF ratio of 38 or lower is very effective in ruling out preeclampsia in the upcoming week. Conversely, higher ratios, such as around 85 or 110, can help identify women who are at a higher short-term risk for the condition [26,27,28]. On this basis, several national and international guidelines, including those of the International Society for the Study of Hypertension in Pregnancy, now recognize angiogenic imbalance as evidence of placental dysfunction and support the use of PlGF-based tests in the evaluation of suspected preeclampsia [29,30,31,32]. VEGF A itself is a key proangiogenic factor involved in placental vascular development, and its interaction with sFlt1 is central to the antiangiogenic state. However, reported VEGF A concentrations in maternal serum are variable and influenced by assay characteristics, binding to sFlt1 and gestational age, and its role as a standalone biomarker is less clearly defined than that of PlGF or the sFlt1/PlGF ratio [18,20,33].

Alongside placental factors, maternal cardiometabolic health modifies both the risk and the phenotype of preeclampsia. Higher pre-pregnancy BMI and excessive gestational weight gain are consistent risk factors, with meta-analyses suggesting that the risk of preeclampsia approximately doubles with every 5 to 7 kg per square meter increase in pre-pregnancy BMI [34,35,36]. Obesity has also been linked to later-onset forms of preeclampsia and may interact with placental dysfunction through chronic low-grade inflammation, insulin resistance and altered adipokine profiles. How maternal BMI relates quantitatively to circulating angiogenic markers in established preeclampsia, however, is less well characterized than the predictive value of these markers themselves [35,37]. There is ongoing interest in integrating clinical risk factors and angiogenic biomarker profiles to better describe the spectrum of preeclampsia and to refine risk stratification in routine care. Many published studies focus on high-risk screening populations or on specific gestational windows, and fewer report combined data on sFlt1, PlGF, VEGF A and the sFlt1/PlGF ratio in women with clinically established preeclampsia compared with uncomplicated pregnancies at a similar gestational stage [38,39].

The present study was designed to compare maternal characteristics and angiogenic markers between women with normal pregnancies and women with preeclampsia. Specifically, we evaluated pre-pregnancy BMI, BMI during pregnancy, gestational age at sampling, and serum concentrations of sFlt1, PlGF, VEGF A, and the sFlt1/PlGF ratio in 30 uncomplicated pregnancies and 60 pregnancies complicated by preeclampsia in the second trimester. Our aim was to describe the pattern of angiogenic imbalance in this cohort and to examine how it relates to maternal anthropometric factors to compare normal and preeclamptic pregnancies.

## 2. Results

A total of 90 pregnant women were included in the analysis, 30 with uncomplicated pregnancies and 60 with preeclampsia. Maternal age was similar in the two groups, with a mean of 29.27 ± 5.90 years in the normal pregnancy group and 29.50 ± 6.74 years in the preeclampsia group (Table 1).

Parity distributions differed descriptively between groups. Among women with normal pregnancies, 11 of 30 (36.7%) were nulliparous, 13 of 30 (43.3%) had one previous birth, 5 of 30 (16.7%) had two previous births, and 1 of 30 (3.3%) had more than two previous births. In the preeclampsia group, 40 of 60 (66.7%) were nulliparous, 16 of 60 (26.7%) had one previous birth, 3 of 60 (5.0%) had two previous births, and 1 of 60 (1.7%) had more than two previous births. More than one prenatal consultation had been attended by 18 of 30 women with normal pregnancies and by 41 of 60 women with preeclampsia, corresponding to 60 percent and 68 percent, respectively.

Body mass index showed clear differences between groups. Mean pre-pregnancy BMI was 22.37 ± 1.03 kg/m^2^ in the normal pregnancy group and 28.15 ± 5.55 kg/m^2^ in the preeclampsia group. BMI during pregnancy was also higher in women with preeclampsia, with means of 26.36 ± 2.67 kg/m^2^ versus 35.18 ± 7.44 kg/m^2^.

Gestational age at enrollment differed by about two weeks between groups. Women with normal pregnancies were included at 23.24 ± 0.81 weeks, whereas those with preeclampsia were enrolled at 25.22 ± 1.24 weeks.

Regarding angiogenic and antiangiogenic markers, sFlt1 concentrations tended to be higher in women with preeclampsia, with a mean of 1322 ± 622.6 pg/mL, compared with 1172 ± 407.2 pg/mL in the normal pregnancy group. In contrast, PlGF levels were markedly lower in preeclampsia, 348.5 ± 171.0 pg/mL, compared with 615.2 ± 269.4 pg/mL in normal pregnancies.

The sFlt1/PlGF ratio very clearly differed between groups. Women with normal pregnancies had a mean ratio of 20.0 ± 4.51, whereas those with preeclampsia had a mean ratio of 60.93 ± 29.30, about three times higher.

VEGF A concentrations were substantially higher in preeclampsia, with a mean of 590.2 ± 432.4 pg/mL compared with 144.4 ± 51.62 pg/mL in the normal pregnancy group. Overall, the pattern of results indicates that PlGF, the sFlt1/PlGF ratio, and VEGF A clearly discriminate between normal and preeclamptic pregnancies in this cohort, while sFlt1 alone shows only a borderline increase in preeclampsia.

Within the preeclampsia group, detailed clinical data were available for all 60 women. Disease onset was before 34 weeks in 26 women (43.3 percent), between 34 and 37 weeks in 28 women (46.7 percent), and at or after 37 weeks in six women (10.0 percent). Exacerbation of hypertension was documented in 6 of 60 women (10.0 percent). A history of chronic hypertension was present in 13 women (21.7 percent). Epigastric pain occurred in 18 women (30.0 percent). Headaches were frequent, reported by 50 of 60 women (83.3 percent). Generalized edema was present in 45 women (75.0 percent). Sudden weight gain of more than 1 kg was recorded in 48 women (80.0 percent). Fetal growth restriction was also reported in 48 women (80.0 percent). Severe complications were less common, with eclampsia in three women (5.0 percent), HELLP syndrome in two women (3.3 percent), and uteroplacental apoplexy in two women (3.3 percent) (Table 2).

**Figure 1:**
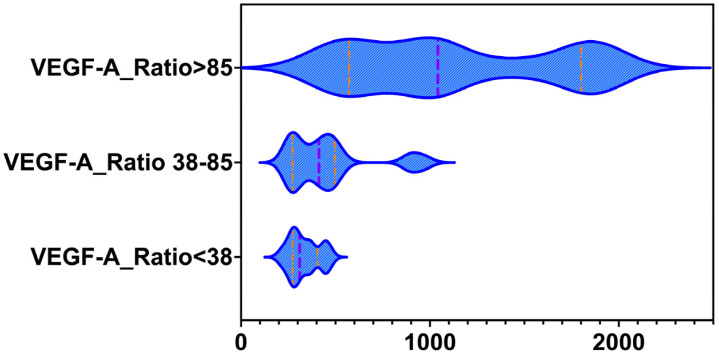
Violin plots of the distribution of VEGF-A. Violin plots showing the distribution of maternal serum VEGF A concentrations after stratification by sFlt1/PlGF ratio cutoffs <38, 38 to 85, and >85. Violin width represents the kernel density of values. Vertical lines indicate the median and the interquartile range. Sample sizes were *n* = 13, 33, and 14, respectively, and the overall difference across groups was significant (*p* < 0.0001).

When the cohort was stratified according to sFlt1-to-PlGF ratio categories, <38, 38–85 and >85, distinct VEGF A patterns were observed, as summarized in Table 3 and Figure 1. In keeping with the overall group means, women with normal pregnancies were concentrated almost entirely in the lowest ratio category, <38, whereas women with preeclampsia were predominantly distributed in the intermediate, 38–85, and high, >85, categories. Across the entire study population, VEGF A concentrations followed the same direction as the sFlt1-to-PlGF ratio. VEGF A values were lowest in the group with a ratio below 38, higher in the 38–85 group, and highest in women with a ratio above 85. Taken together, the distribution shows that higher sFlt1/PlGF ratio categories, which indicate a more significant angiogenic imbalance, are linked to higher circulating VEGF A levels. In contrast, low ratio values, mostly seen in uncomplicated pregnancies, are associated with lower VEGF A levels.

**Figure 2:**
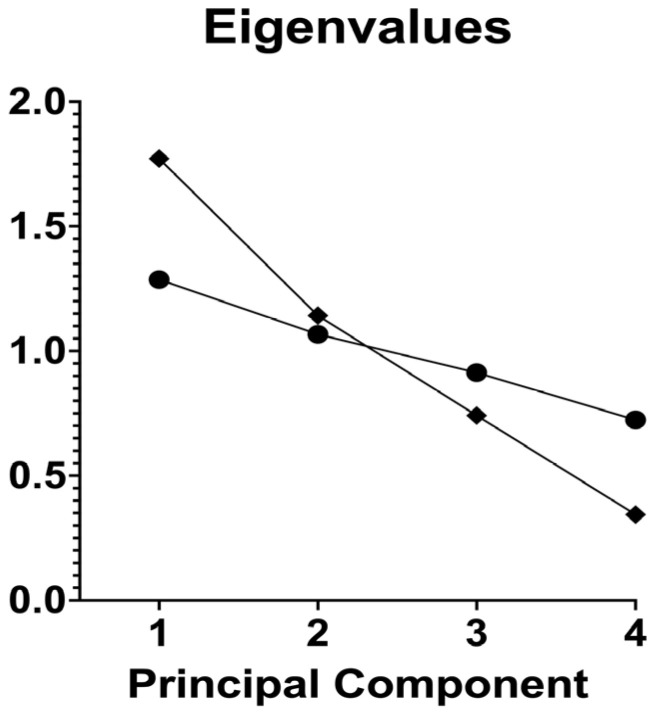
This figure presents a scree plot of the PCA eigenvalues across the four principal components. The observed eigenvalues (diamonds) decline from PC1 (about 1.77) to PC4 (about 0.34), with the steepest drop occurring between PC2 and PC3. The plot also shows the eigenvalues expected under parallel analysis (circles). PC1 and PC2 exceed the corresponding parallel analysis values, whereas PC3 and PC4 fall below them, indicating that the meaningful structure in the data is mainly captured by the first two components.

Principal component analysis was performed on four maternal variables in the preeclampsia group: age, pre-pregnancy BMI, BMI during pregnancy, and gestational age at sampling, with 60 cases included (Table 4 and Figure 2). The scree plot showed a clear drop after the first component. PC1 had an eigenvalue of 1.772 and explained 44.3% of the total variance. PC2, PC3, and PC4 had eigenvalues of 1.142, 0.742, and 0.344, explaining 28.6%, 18.6%, and 8.6% of variance, respectively. Parallel analysis using 1000 simulations indicated that only PC1 should be retained, since only PC1 exceeded the eigenvalues expected from random data. Loadings on PC1 were highest for pre-pregnancy BMI (0.849) and BMI during pregnancy (0.862), with a moderate contribution from gestational age (0.500) and a smaller contribution from age (0.241).

In the preeclampsia group (*n* = 60), a multiple linear regression model was fitted with maternal VEGF A as the dependent variable and sFlt1, PlGF, the sFlt1 PlGF ratio, and PC1 (from the PCA of maternal characteristics) as predictors, Table 5. The overall model was not significant, F(4,55) = 1.476, *p* = 0.222, and explained 9.7% of the variance in VEGF A (R^2^ = 0.0969). Among predictors, sFlt1 showed a small positive association with VEGF A (β = 0.1828, 95% CI 0.000217 to 0.3654, *p* = 0.0497). PlGF (β = 0.4138, 95% CI −0.2537 to 1.081, *p* = 0.219), the sFlt1-to-PlGF ratio (β = −0.8636, 95% CI −4.681 to 2.954, *p* = 0.652), and PC1 (β = −57.42, 95% CI −142.4 to 27.55, *p* = 0.181) were not significant predictors. Multicollinearity was negligible (VIF 1.01 to 1.06). Residual diagnostics indicated deviation from normality (D’Agostino–Pearson, Anderson–Darling, Shapiro–Wilk, and Kolmogorov–Smirnov tests, all *p* < 0.0001).

## 3. Discussion

In this cross-sectional study of 30 uncomplicated pregnancies and 60 pregnancies complicated by preeclampsia, we found that preeclampsia was associated with higher pre-pregnancy and antenatal BMI, slightly later gestational age at sampling, and a marked angiogenic imbalance, characterized by low PlGF, a substantially increased sFlt1/PlGF ratio, and higher VEGF A concentrations. In contrast, maternal age did not differ, and sFlt1 alone showed only a borderline increase. These findings are broadly consistent with current concepts of preeclampsia as a disorder that combines placental malperfusion and systemic endothelial dysfunction with maternal cardiometabolic susceptibility [18,40].

The difference in BMI between groups was large, almost 6 kg/m^2^ for pre-pregnancy BMI and nearly 9 kg/m^2^ for BMI during pregnancy. This pattern aligns with extensive epidemiological data showing that increasing maternal adiposity is a strong, graded risk factor for preeclampsia. Systematic reviews and meta-analyses, including more than one million pregnancies, have shown that the risk of preeclampsia approximately doubles with each 5–7 kg/m^2^ increase in pre-pregnancy BMI [34,41]. More recent work continues to support a dose–response association between higher BMI and hypertensive disorders of pregnancy [42,43]. Our results are in line with these observations and suggest that, in this cohort, women who developed preeclampsia entered pregnancy with a substantially less favorable anthropometric profile and accumulated more weight during gestation. Mechanistically, obesity-related chronic low-grade inflammation, insulin resistance, and altered adipokine secretion may amplify placental and endothelial stress, thereby lowering the threshold at which placental antiangiogenic signals clinically manifest as preeclampsia [41,42]. Because we did not perform multivariable modeling, we cannot determine whether the observed differences in angiogenic markers are independent of BMI. Still, the clear anthropometric separation between groups underscores the need to consider maternal size when interpreting biomarker levels in clinical practice and in future studies.

The angiogenic marker profile observed here, low PlGF and a markedly elevated sFlt1/PlGF ratio in preeclampsia, closely fits with the now well-established model of angiogenic imbalance in this disorder. Numerous studies have reported increased circulating sFlt1 and reduced PlGF in women with established preeclampsia, as well as in the weeks preceding clinical onset [40,44,45]. The ratio of these two markers amplifies their opposing changes and has repeatedly been shown to outperform either marker alone for diagnosis and short-term prediction [46,47,48,49,50,51]. In our cohort, PlGF was approximately halved in preeclampsia compared with normal pregnancy, and the sFlt1-to-PlGF ratio was approximately threefold higher.

In contrast, sFlt1 alone showed only a borderline higher mean in preeclampsia, with a *p*-value just above the conventional 0.05 threshold. This pattern is plausible for several reasons. First, sFlt1 concentrations display wide interindividual variation, and group differences may be harder to detect than for PlGF, which tends to be more tightly distributed [40,52]. Second, our measurements were obtained in the mid-second trimester, around 23–25 weeks, a gestational window in which sFlt1 levels in normal pregnancy are relatively low and begin to rise more steeply only later in gestation, whereas PlGF is already high and close to its peak [52]. Several longitudinal studies suggest that the most pronounced divergence in sFlt1 between women who will develop preeclampsia and controls occurs in the few weeks immediately preceding disease onset, particularly in early-onset and severe cases [40,53]. Third, our preeclampsia group was clinically heterogeneous with respect to time of onset and severity, which may have diluted differences in sFlt1 when all cases were analyzed together. These considerations suggest that, in a cross-sectional design with a single early second-trimester measurement, the sFlt1-to-PlGF ratio, driven largely by low PlGF levels, may be more informative than sFlt1 alone, in agreement with the current literature [46,49,54].

The behavior of VEGF A in our study warrants specific comment. We found higher serum VEGF A concentrations in women with preeclampsia compared to controls, whereas many early studies, including the pivotal work by Levine et al., reported lower free VEGF levels in women with preeclampsia, which was interpreted as resulting from sequestration by excess circulating sFlt1 [40]. Several factors may explain this apparent contradiction. Firstly, reported VEGF levels in preeclampsia are highly dependent on the assay used. Some methods measure only free VEGF, while others detect total immunoreactive VEGF, including VEGF bound to sFlt1 or other proteins [24,33,55]. Studies employing assays for total VEGF or different sample types have sometimes observed higher, rather than lower, VEGF levels in preeclampsia or in more severe cases, possibly reflecting increased production by activated endothelial cells and leukocytes in response to systemic inflammation and hypoxia [33,56]. Second, our cohort included a large proportion of women with early- or moderately preterm-onset and frequent fetal growth restriction, a phenotype often linked to more significant placental hypoxia, which may promote upregulation of VEGF transcripts and protein in certain compartments despite concurrent binding by sFlt1 [18,24,56]. Lastly, the relatively high variability we observed in VEGF A levels suggests that random errors and unmeasured confounders may also be involved. Taken together, our findings support the view that VEGF A measured in maternal serum is a complex and context-dependent marker, and its standalone diagnostic value in preeclampsia appears less consistent than that of PlGF or the sFlt1-to-PlGF ratio, consistent with recent reviews [24,33,51].

VEGF A warrants careful interpretation in the context of preeclampsia, given its dependence on contextual factors for both its biology and measurement. Excess placental sFlt1 acts as a soluble decoy receptor, binding to VEGF and PlGF. This binding reduces the availability of ligands for endothelial receptors, contributing to an antiangiogenic phenotype characterized by endothelial dysfunction, even as total circulating ligand production increases in response to hypoxia or inflammation. Recent mechanistic and clinical reviews emphasize that the detectable VEGF A signal can derive from different sources, such as free versus total immunoreactive ligands, and that the outputs of the VEGF pathway are also influenced by isoform composition and receptor context. Consequently, higher measured levels of VEGF A do not necessarily indicate preserved proangiogenic signaling. Moreover, serum VEGF quantification is particularly sensitive to preanalytical conditions, including the release of factors related to platelets during clotting and handling, which can exacerbate interindividual variability [24,57,58,59,60]. Recent integrative mechanistic reviews frame preeclampsia as a system-level angiogenic imbalance in which placentally derived sFlt1 sequesters both VEGF and PlGF, so elevated circulating VEGF A should be interpreted alongside the sFlt1, PlGF axis and ligand bioavailability rather than as an isolated proangiogenic signal [24,60,61]. Emerging work also extends beyond individual biomarkers by integrating broader endothelial and angiolymphatic signatures to relate VEGF, sFlt1, and PlGF interactions to placental pathology and clinical severity [58].

The clinical profile of the preeclampsia group is also informative. Nearly half of the women had onset of disease before 34 weeks, and another large proportion between 34 and 37 weeks. Fetal growth restriction, sudden weight gain, generalized edema, and headaches were very common, whereas eclampsia, HELLP syndrome, and uteroplacental apoplexy were rare. This distribution suggests a cohort with predominantly preterm, placenta-mediated disease, but with relatively effective antenatal monitoring and intervention that may have mitigated progression to the most severe maternal complications. Early-onset and growth-restricted forms of preeclampsia are strongly linked to placental malperfusion and display more pronounced angiogenic imbalance than late-onset forms, particularly for the sFlt1/PlGF ratio [40,50,54,62]. The marked elevation of the sFlt1/PlGF ratio in our preeclampsia group, therefore, matches the clinical composition of the cohort.

From a translational perspective, our findings reinforce current recommendations that angiogenic biomarkers can meaningfully supplement clinical assessment in suspected preeclampsia. Large prospective studies, notably PROGNOSIS and subsequent validation cohorts, have shown that an sFlt1-to-PlGF ratio at or below 38 has a very high negative predictive value for ruling out preeclampsia in the following week, while higher cutoffs can help identify women at increased short-term risk of adverse outcomes [46,47,48,49,63]. The clinical sFlt1, PlGF ratio cutoff of 38 was validated in women with suspected preeclampsia as a short-term rule-out threshold, and its use is context dependent. Since both sFlt1 and PlGF change with gestational age, the later sampling in the preeclampsia group could shift the ratio upward and affect classification around fixed thresholds. Accordingly, we use these cut points for descriptive stratification only, and we avoid interpreting them as validated diagnostic cutoffs in this cohort and assay setting [48]. Recent national guidelines now recommend the use of PlGF-based tests or the sFlt1/PlGF ratio, where available, in evaluating women with suspected preeclampsia [29,46,64,65,66]. Although our study did not assess predictive performance or define cutoffs, the large separation we observed in the sFlt1-to-PlGF ratio between uncomplicated and preeclamptic pregnancies at 23–25 weeks supports the biological plausibility of these guidelines in a real-world cohort.

Several limitations should be acknowledged. The study sample is small and unbalanced, with twice as many preeclampsia cases as controls, which may reduce accuracy, especially for variables with high variability such as sFlt1 and VEGF A. The design is cross-sectional with only one measurement mid-gestation, so it cannot evaluate changes over time or the predictive value of biomarkers. Gestational age at sampling was about two weeks later in the preeclampsia group, and since both sFlt1 and the sFlt1-to-PlGF ratio usually increase with advancing gestation, while PlGF peaks and then declines, some of the between-group difference may reflect normal gestational trends rather than disease alone [40,52]. Residual confounding by maternal pre-pregnancy BMI and gestational age at sampling cannot be excluded. Because of the limited sample size, we did not perform a comprehensive multivariable adjustment, and the observed between-group differences should be interpreted with these factors in mind. Because this was a cross-sectional, single-time-point analysis performed after diagnosis, our findings are limited to associations and do not support causal conclusions about BMI or angiogenic pathways in preeclampsia. The design also precludes evaluating temporal trajectories or estimating predictive performance for future disease. Lastly, our data are from a single center, and the ability to apply these findings to other populations, especially those with lower BMI or mainly late-onset preeclampsia, is uncertain.

Despite these constraints, this study provides a coherent picture in which higher maternal BMI, earlier and more severe forms of preeclampsia, and a strong angiogenic imbalance, dominated by low PlGF and a high sFlt1-to-PlGF ratio, cluster together mid-gestation. This pattern mirrors current mechanistic models and large multicenter studies. It suggests that, even when measured at a single time point, combined angiogenic markers can help distinguish uncomplicated pregnancies from those with preeclampsia more effectively than sFlt1 alone. At the same time, maternal anthropometric factors remain important background determinants of risk.

## 4. Materials and Methods

### 4.1. Study Design and Participants

This was an observational analytical study encompassing 90 pregnant women in their second trimester, comprising 30 cases of uncomplicated pregnancies and 60 cases complicated by preeclampsia. All participants had singleton gestations.

Women with uncomplicated pregnancies had no history of chronic hypertension, renal or hepatic disease, diabetes mellitus, autoimmune disease, or other significant medical conditions, and remained normotensive with proper fetal growth throughout pregnancy.

Women in the preeclampsia group were consecutively enrolled at the time of diagnosis or referral for preeclampsia evaluation. Exclusion criteria were multiple pregnancy, known fetal congenital malformations, chronic kidney or liver disease, pregestational diabetes, clinically evident infection, and hematologic or systemic autoimmune disease.

The protocol was approved by the local institutional ethics committee, complied with the Declaration of Helsinki, and was approved by the Ethics Committee of the Clinical Municipal Hospital Filantropia Craiova (no. approval no. 7932/7 April 2025), Dolj, Romania. All participants provided written informed consent before enrollment.

### 4.2. Diagnosis and Classification of Preeclampsia

Preeclampsia was defined as new-onset hypertension after 20 weeks of gestation in a previously normotensive woman, associated with proteinuria or maternal organ dysfunction, in accordance with contemporary recommendations of the American College of Obstetricians and Gynecologists and the International Society for the Study of Hypertension in Pregnancy [67].

Hypertension was defined as a systolic blood pressure of at least 140 mmHg and/or a diastolic blood pressure of at least 90 mmHg, measured on at least two occasions using an appropriately sized cuff after a period of rest in the sitting position [68]. Proteinuria was defined as a protein excretion of at least 300 mg per 24 h, a protein-to-creatinine ratio of at least 30 mg/mmol, or a dipstick result of at least 1 plus when more precise methods were not available.

Maternal organ involvement was considered present in the case of any of the following, in the absence of other obvious causes, according to international guidelines: thrombocytopenia, elevated liver enzymes with right upper quadrant or epigastric pain, new-onset neurological symptoms such as persistent headache or visual disturbances, pulmonary edema, or new-onset renal impairment.

Early-onset preeclampsia was defined as clinical onset before 34 weeks of gestation and late-onset preeclampsia as onset at or after 34 weeks. Preterm preeclampsia was defined as the onset or indication for delivery before 37 weeks. In the preeclampsia group, the presence of HELLP syndrome, eclampsia and uteroplacental apoplexy, as well as fetal growth restriction, was recorded based on standard clinical and ultrasound criteria.

Women in the uncomplicated pregnancy group were normotensive throughout gestation, had no proteinuria, and delivered appropriate-for-gestational-age neonates at term.

### 4.3. Clinical and Obstetric Data

At inclusion, all women underwent a structured clinical and obstetric assessment. PE pregnancies were managed by a multidisciplinary team comprising an obstetrician, a nephrologist, an anesthesiologist, and a neonatologist. Maternal age, parity, previous obstetric history, and number of antenatal consultations attended were recorded. Height and pre-pregnancy weight were obtained from medical records or early pregnancy documentation and used to calculate pre-pregnancy body mass index (BMI, as weight in kilograms divided by height in meters squared). The current weight at the time of blood sampling was measured and used to calculate BMI during pregnancy.

Gestational age was determined from the last menstrual period and confirmed or corrected by first-trimester ultrasound. For the present analysis, only women sampled in the second trimester were included. Gestational age at sampling was calculated in completed weeks.

### 4.4. Blood Sampling and Laboratory Analysis

Peripheral venous blood was drawn from each participant in the morning, after a short period of rest, at the time of the second trimester visit. All samples were obtained before the initiation of antihypertensive or magnesium sulfate therapy in women with newly diagnosed preeclampsia.

Blood was collected into serum separator tubes, allowed to clot at room temperature, and centrifuged according to standard procedures. The serum was aliquoted into labeled cryovials and stored at low temperature until batch analysis to avoid repeated freeze–thaw cycles, following protocols similar to those used in previous studies of angiogenic and oxidative stress mediators in preeclampsia.

Serum concentrations of sFlt-1 (Cat. No.: E-EL-H6117; sensitivity: 4.69 pg/mL; detection range: 7.81–500 pg/mL; there was no notable cross-reactivity or interference identified between human sVEGFR-1 and its analogues; recovery 80–120%; detection method: colorimetric method, ELISA, sandwich), PlGF (Cat. No.: E-EL-H1555; sensitivity: 0.63 pg/mL; detection range: 1.96–125 pg/mL; recovery 80–120%; no significant cross-reactivity or interference was observed between human PGF and its analogues; detection method: colorimetric method, ELISA, sandwich), and VEGF A (Cat. No.: E-EL-H0111; sensitivity: 18.75 pg/mL; detection range: 31.25–2000 pg/mL; there was no significant cross-reactivity or interference between human VEGF-A and its analogs observed; recovery 80–120%; detection method: colorimetric method, ELISA, sandwich) were measured using commercially available enzyme-linked immunosorbent assay kits, ELISAs, in accordance with the manufacturer’s instructions; if necessary, the samples were diluted accordingly. The sFlt1-to-PlGF ratio was calculated for each participant by dividing the sFlt1 concentration by the PlGF concentration expressed in the same units.

### 4.5. Statistical Analysis

Continuous variables were inspected for outliers and tested for normal distribution using the D’Agostino–Pearson omnibus test, following the approach used in related work. Variables with an approximately Gaussian distribution are presented as mean plus or minus standard deviation, whereas skewed variables are summarized as median and interquartile range. Categorical variables are presented as counts and percentages.

The main objective of the analysis was to compare maternal characteristics, gestational age at sampling, and angiogenic markers between women with uncomplicated pregnancies and those with preeclampsia. A *p*-value below 0.05 was considered statistically significant. Statistical analyses were carried out using GraphPad Prism software version 10.6.1 (GraphPad Software, San Diego, CA, USA).

## 5. Conclusions

In this cohort of women sampled in the second trimester, pregnancies complicated by preeclampsia were characterized by higher pre-pregnancy BMI and higher BMI during pregnancy, compared with uncomplicated pregnancies, while maternal age was similar between groups. This supports the concept that maternal adiposity is an important background risk factor for preeclampsia.

At the biomarker level, preeclampsia was associated with a clear angiogenic imbalance. PlGF concentrations were significantly reduced, and the sFlt1-to-PlGF ratio was markedly increased in preeclamptic pregnancies, whereas sFlt1 alone showed only a borderline elevation. VEGF A concentrations were also higher in the preeclampsia group, although with considerable variability. Taken together, these findings indicate that, in mid-gestation, combined angiogenic markers, particularly the sFlt1-to-PlGF ratio, more robustly discriminate between normal and preeclamptic pregnancies than sFlt1 alone.

Clinically, many women with preeclampsia in this study had early- or preterm-onset and frequent fetal growth restriction, consistent with a predominantly placenta-mediated phenotype and with the pronounced angiogenic imbalance observed. The cross-sectional design, modest sample size, and single-center setting limit generalizability and preclude assessment of predictive performance. However, the pattern identified, higher BMI combined with low PlGF, high sFlt1-to-PlGF ratio, and increased VEGF A, is coherent with current pathophysiological models and supports the integration of angiogenic markers into the evaluation of women with suspected preeclampsia, alongside careful clinical and anthropometric assessment.

## Figures and Tables

**Table 1:** Maternal characteristics and angiogenic markers in women with normal pregnancy and preeclampsia.

		N Pregnancy (*n* = 30)		PE Pregnancy (*n* = 60)		*p*-Value
Parity						
Nulliparous		11		40		
1 childbirth		13		16		
2 childbirth		5		3		
>2 childbirth		1		1		
Prenatal consult (>1)		Yes	No	Yes	No	
		18	12	41	19	
Age	Mean ± SD	29.27 ± 5.90		29.50 ± 6.74		0.872
BMI pre-pregnancy	Mean ± SD	22.37 ± 1.03		28.15 ± 5.55		<0.0001 *
BMI during pregnancy	Mean ± SD	26.36 ± 2.67		35.18 ± 7.44		<0.0001 *
Gestational age at enrollment (weeks)	Mean ± SD	23.24 ± 0.81		25.22 ± 1.24		<0.0001 *
s-Flt	Mean ± SD	1172 ± 407.2		1322 ± 622.6		0.236
PIGF	Mean ± SD	615.2 ± 269.4		348.5 ± 171		<0.0001 *
s-Flt/PIGF ratio	Mean ± SD	20 ± 4.51		60.93 ± 29.30		<0.0001 *
VEGF-A	Mean ± SD	144.4 ± 51.62		590.2 ± 432.4		<0.0001 *

Data are presented as mean ± standard deviation or *n* (percent). BMI, body mass index; sFlt1, soluble fms-like tyrosine kinase 1; PlGF, placental growth factor; VEGF-A, vascular endothelial growth factor A. *: reached statistical significance.

**Table 2:** Clinical features and complications in women with preeclampsia.

Preeclampsia				
Debut of PE (*n*)	<34 weeks	34–37 weeks		37 weeks
	26	28		6
Exacerbation of HTN (*n*)	Yes		No	
	6		54	
Epigastric pain (*n*)	Yes		No	
	18		42	
Headaches (*n*)	Yes		No	
	50		10	
Generalized edema (*n*)	Yes		No	
	45		15	
Sudden weight gain >1kg (*n*)	Yes		No	
	48		12	
RCIU (*n*)	Yes		No	
	48		12	
HTN (*n*)	Yes		No	
	13		47	
Eclampsia (*n*)	Yes		No	
	3		57	
HELLP syndrome (*n*)	Yes		No	
	2		58	
Uteroplacental apoplexy (*n*)	Yes		No	
	2		58	

Data are presented as *n* (%) of women with the specified sign, symptom or complication.

**Table 3:** VEGF A distribution after sFlt1/PlGF ratio.

	VEGF A Ratio <38	VEGF A Ratio 38–85	VEGF A Ratio >85	*p*-Value
Number of values	13	33	14	<0.0001 *
Minimum	215.0	253.7	440.7	
25% Percentile	272.7	271.9	571.7	
Median	310.6	412.7	1042	
75% Percentile	405.1	496.4	1801	
Maximum	469.8	975.8	1892	
Range	254.8	722.2	1451	

VEGF-A distribution regarding the sFlt1/PlGF ratio cutoffs; *: reached the statistical significance.

**Table 4:** Principal component analysis.

	**PC1**	**PC2**	**PC3**	**PC4**
Eigenvalue	1.772	1.142	0.7419	0.3439
Proportion of variance	44.30%	28.56%	18.55%	8.60%
Cumulative proportion of variance	44.30%	72.85%	91.40%	100.00%
**Loadings**				
Variable	Age-PE	BMI-preP-PE	BMI-duringP-PE	Gestational age-PE
PC1 loading	0.241	0.849	0.862	0.500

**Table 5:** Multiple linear regression.

Model					
Analysis of Variance	SS	DF	MS	F (DFn, DFd)	*p*-value
Regression	1,069,262	4	267,315	F (4, 55) = 1.476	0.2221
s-Flt-PE	729,220	1	729,220	F (1, 55) = 4.026	0.0497 *
PlGF-PE	279,554	1	279,554	F (1, 55) = 1.543	0.2194
s-Flt/PlGF ratio-PE	37,236	1	37,236	F (1, 55) = 0.2056	0.6520
PC1	332,211	1	332,211	F (1, 55) = 1.834	0.1812
Parameter estimates	Variable	Estimate	95% CI (profile likelihood)	|t|	*p*-value
β0	Intercept	257.1	−166.9 to 681.1	1.215	0.2295
β1	s-Flt-PE	0.1828	0.0002171 to 0.3654	2.006	0.0497 *
β2	PlGF-PE	0.4138	−0.2537 to 1.081	1.242	0.2194
β3	s-Flt/PlGF ratio-PE	−0.8636	−4.681 to 2.954	0.4534	0.6520
β4	PC1	−57.42	−142.4 to 27.55	1.354	0.1812

SS: sum of squares; DF: degrees of freedom; MS: mean square; DFn: numerator DF; DFd: denominator DF; *: reached the statistical significance.

## Data Availability

The data used to support the findings of this study are available from the corresponding author upon reasonable request, within 6 months.
